# Evolutionary Psychology of Eating Disorders: An Explorative Study in Patients With Anorexia Nervosa and Bulimia Nervosa

**DOI:** 10.3389/fpsyg.2018.02122

**Published:** 2018-10-31

**Authors:** Johanna Nettersheim, Gabriele Gerlach, Stephan Herpertz, Riadh Abed, Aurelio J. Figueredo, Martin Brüne

**Affiliations:** ^1^LWL University Hospital Bochum, Department of Psychiatry, Psychotherapy and Preventive Medicine, Division of Cognitive Neuropsychiatry, Ruhr University Bochum, Bochum, Germany; ^2^LWL University Hospital Bochum, Department of Psychosomatic Medicine, Ruhr University Bochum, Bochum, Germany; ^3^Mental Health Tribunals, Ministry of Justice, Sheffield, United Kingdom; ^4^Department of Psychology, School of Mind, Brain, and Behavior, College of Science, University of Arizona, Tucson, AZ, United States

**Keywords:** eating disorders, anorexia nervosa, bulimia, life history strategy, executive functioning, mate value, intrasexual competition

## Abstract

Prior research on non-clinical samples has lent support to the sexual competition hypothesis for eating disorders (SCH) where the drive for thinness can be seen as an originally adaptive strategy for women to preserve a nubile female shape, which, when driven to an extreme, may cause eating disorders. Restrictive versus impulsive eating behavior may also be relevant for individual differences in allocation of resources to either mating effort or somatic growth, reflected in an evolutionary concept called “Life History Theory” (LHT). In this study, we aimed to test the SCH and predictions from LHT in female patients with clinically manifest eating disorders. Accordingly, 20 women diagnosed with anorexia nervosa (AN), 20 with bulimia nervosa (BN), and 29 age-matched controls completed a package of questionnaires comprising measures for behavioral features and attitudes related to eating behavior, intrasexual competition, life history strategy, executive functioning and mating effort. In line with predictions, we found that relatively faster life history strategies were associated with poorer executive functioning, lower perceived own mate value, greater intrasexual competition for mates but not for status, and, in part, with greater disordered eating behavior. Comparisons between AN and BN revealed that individuals with BN tended to pursue a “fast” life history strategy, whereas people with AN were more similar to controls in pursuing a “slow” life history strategy. Moreover, intrasexual competition for mates was significantly predicted by the severity of disordered eating behavior. Together, our findings lend partial support to the SCH for eating disorders. We discuss the implications and limitations of our study findings.

## Introduction

Anorexia nervosa (AN) and Bulimia nervosa (BN) are diagnostic categories of eating disorders according to ICD-10 and DSM-5 classifications. The two conditions share core features of morbid fear of fatness, distorted body image and a pattern of behavior aimed at weight reduction including purging, restriction of food intake or excessive exercise (World Health Organization, 1993; American Psychiatric Association, 2013). AN is characterized by low body weight whereas BN is associated with binge eating and a normal or even raised body weight.

Eating disorders can prove difficult to treat with 50% remission rates for AN and 75% for BN at 10 years follow up (Keel and Brown, 2010). Also, AN is considered to have the highest mortality rate among mental disorders (Harris and Barraclough, 1998) with 5.9% of cases resulting in a fatal outcome (Fichter and Quadflieg, 2016).

This clinically unsatisfactory state of affairs results, at least in part, from the fact that the etiology of eating disorders remains poorly understood.

Evidence suggests at least modest heritability (Yilmaz et al., 2015; Bulik et al., 2016). Furthermore, neurobiologically, dysfunctions of the serotonergic and neuropeptide systems seem to play a role (Kaye, 2008). Psychosocial factors involved in BN include weight concerns, negative body image and low self-esteem (Jacobi, 2005).

The most notable feature of the epidemiology of EDs, including AN and BN, is their marked female preponderance with a female-to-male sex ratio of 10:1 or greater (Gordon, 1990; Hudson et al., 2007). Also, both AN and BN are by far more prevalent in developed countries compared to developing countries, particularly when considering sub-threshold phenotypes (Katzman et al., 2004). Put another way, the paradox here is that the incidence and prevalence of eating disorders is much higher in cultural environments where high-energy nutrition abound (Nesse, 2017). It should be noted, however, that eating disorders occur worldwide and tend to increase in non-Western-countries most probably in association with industrialization, urbanization, and westernization (Pike et al., 2014; Erskine et al., 2016).

### Evolutionary Theories for Eating Disorders

A number of evolutionarily-informed theories and hypotheses have been proposed. For example, the “Reproductive Suppression Hypothesis” of AN considers eating restriction as an unconscious strategy to delay reproduction in times of disadvantageous environmental conditions by lowering the amount of body fat to a level incompatible with ovulation (Wasser and Barash, 1983; Surbey, 1987; Crawford, 1989; Voland and Voland, 1989; Condit, 1990; Salmon et al., 2008). Along similar lines, Salmon et al. (2008) proposed that cultural, social, and ecological factors such as enhanced female–female competition or unwanted sexual attention from males in modern urbanized Western societies may cause fears related to weight gain and body image (Salmon et al., 2008). They found that high stress conditions in five different stressful scenarios (i.e., exposure to female competition, male attention, media) induced greater body dissatisfaction and anorexic-type behavior in women compared to low stress conditions (Salmon et al., 2008). Consistent with the Reproductive Suppression Hypothesis, another study reported that women who perceive low levels of support, particularly from romantic partners and family, are prone to dieting and do not feel ready for parenthood, suggesting that reproductive suppression in times of poor environmental conditions is causal in the development of AN (Juda et al., 2004).

An alternative evolutionary approach in relation to the Reproductive Suppression Hypothesis was put forth by Mealey (2000) who considered AN as the consequence of a “manipulative strategy imposed on subordinates by dominants” (Mealey, 2000), whereby subordinate females are reproductively suppressed by dominants.

Other evolutionary hypotheses have posited that symptoms of AN may help to cope with famine, whereby food restriction, denial of starvation and hyperactivity could represent an adaptive behavior that helped ancestral nomadic foragers to migrate from depleted environments to more promising surroundings in times of food shortages (Guisinger, 2003).

Another quite speculative hypothesis proposed that AN could have evolved as a response to perceived social threat, for instance, the exclusion from one’s social group, where resuming normal eating habits could indicate re-entering competition for status (Gatward, 2007).

These evolutionary approaches are limited in their explanatory power, as they focus exclusively on AN where food restriction causes low body weight, which in turn can lead to amenorrhoea and reproductive suppression, whereas this does not occur in BN.

### The Sexual Competition Hypothesis

An alternative evolutionary model that attempts to the explain the whole spectrum of eating disorders including AN and BN is the “Sexual Competition Hypothesis” (SCH) (Abed, 1998). Intrasexual competition is relevant to both males and females, yet the behavioral expressions differ between the sexes. While males compete among themselves through the display of status, resources, and dominance (Puts, 2010; Arnocky and Carré, 2016), females tend to act more often through strategies such as self-promotion (including displays of physical attractiveness) and the derogation of rivals (Vaillancourt, 2013). These different strategies are suggested to have evolved, because males and females differ in the traits that increase their mate value and therefore help them to attract members of the other sex. Whereas females appreciate features such as earning capacity, ambition, material wealth and status of potential mates, males prefer mates with a high degree of reproductive potential, physical attractiveness, and chastity (Buss, 1989; Buss and Schmitt, 1993; Singh, 1993; Shackelford et al., 2002; Li et al., 2011).

Hence, in line with this reasoning the SCH proposes that female Intra-sexual competition is the biological root for the drive for thinness, an adaptive response originally suited to the ancestral environment and, that the extreme version of this manifests itself in what we know as eating disorders. The SCH proposes that the whole range of eating disorders (primarily AN and BN) are manifestations of abnormally intense female intra-sexual competition whereby autonomous females of reproductive age compete with each other in the novel environment of modern (western and westernized) cities through a strategy of “the pursuit of thinness” as a signal of youth (see below). This is claimed to lead to a state of “runaway female intra-sexual competition” the extreme version of which is eating disorders (Abed, 1998).

The SCH is based on the fact that throughout human evolutionary history the female shape has been a reliable indicator of the female’s reproductive history and consequently her reproductive potential (Singh, 1993; Bovet and Raymond, 2015). Youth and good health have always been the major determinants of female mate value not least because of the finite reproductive window in humans that abruptly ends with menopause (Buss, 1987). The visual signal for a female’s peak reproductive potential in the ancestral environment was the female nubile shape which was generally short-lived and deteriorated with the repeated cycles of gestation and lactation (Symons, 1979, 1995).

It is suggested that in advanced western societies good health has become ubiquitous and thus has declined in importance as a competitive signal (Abed, 1998). Hence, according to SCH, signaling a youthful appearance became the primary means of female intra-sexual competition (Abed, 1998). However, studies have found that thinness in females (especially in affluent societies) is consistently associated with youthfulness (Singh, 1993, 1994a,b). Hence, according to SCH, female intrasexual competition in affluent westernized societies became focused on the preservation and/or the recreation of the nubile shape through a strategy of the pursuit of thinness to display signs of youth. The SCH further proposes that other important factors serve to upregulate the intensity of female intra-sexual competition. Some of the major factors include: (a) female autonomy that involves the ability of females to make mating decisions with relatively little interference from kin. This contrasts sharply with the situation during the majority of human evolutionary history (as well as in present-day traditional societies) where most of the important female mating decisions were taken by parents and elders (Dickemann, 1981; Apostolou, 2007). This means that in the modern western environment females must compete largely through their own efforts. (b) Living in cities where abnormally large numbers of autonomous females live in close proximity to each other. (c) Reduced fertility (birth rates) (Vining, 1986) has meant that females have increasingly managed to preserve their nubile shape beyond the age of nubility thus creating the novel phenomena of the pseudo-nubile female which further intensifies female intra-sexual competition (Abed, 1998). (d) The ubiquity of abnormally attractive youthful nubile female images in the media that are mistaken for competitors (Ferguson et al., 2011).

Therefore, the SCH is based on the idea that there is a mismatch between the design of the female’s psychological adaptations for mate attraction and retention and for competing with rival females on the one hand and the novel circumstances of the modern westernized urban environment on the other.

The basis for considering the SCH as a common etiological framework for AN and BN include the fact that these conditions share the extreme female preponderance referred to above as well as sharing the core psychopathological features of preoccupation with body shape and weight and a pattern of behavior aimed at weight reduction (World Health Organization, 1993; American Psychiatric Association, 2013). In addition, the line of demarcation between these two conditions can be clinically hazy as many patients move between the diagnostic categories over time with estimates of 15% from BN to AN and about 30% from AN to BN (Eddy et al., 2008).

Hence, the SCH claims to offer an explanation for the phenomenon of the drive for thinness, the marked female preponderance, the fact that these disorders affect primarily females of reproductive age and also offers an explanation of the geographical distribution of these disorders. Further elaborations of this hypothesis suggested a distinction between intrasexual competition for mates (which is involved in both AN and BN according to SCH) and intrasexual competition for status (primarily involved in AN, based on the findings of Faer et al., 2005). However, the findings from Faer et al. (2005) study showed that both anorexic and bulimic patterns of abnormal eating behavior in their non-clinical population were driven by intrasexual competition for mates and that, although competition for status was higher in those with the anorexic tendencies, this was secondary to competition for mates.

Hence, if AN and BN are associated with abnormally intense intrasexual competition as the “Sexual Competition Hypothesis” proposes, the question arises as to what factors lead to the diverse variety of anorexic and bulimic presentations seen in clinical practice? One promising avenue of enquiry was to consider the role of different life history strategies in shaping and influencing the manifestations of female intrasexual competition (Abed et al., 2012).

### Life History Theory

Life History Theory (LHT) posits that organisms allocate resources to either somatic effort or reproduction (Stearns, 1992; Kaplan and Gangestad, 2005; Ellis et al., 2009; Flatt and Heyland, 2011). S*omatic effort* includes *growth*, *survival* or *body maintenance*, whereas *reproductive effort* entails *mating effort*, *parenting effort* and *nepotistic effort*, each of which is offset or delayed in favor of the other (Geary, 2002; Del Giudice, 2014). Specifically, *mating effort* comprises obtaining and retaining mates, whereas *parental effort* comprises producing and rearing the resulting offspring (Figueredo et al., 2006; Abed et al., 2012; Figueredo et al., 2012). These trade-offs generate different life history strategies between and within species that are not functionally independent of one another, but form a general pattern of trait covariation that can be aligned on a one-dimensional scale ranging from “slow” to “fast” life history patterns (Ellis et al., 2009; Jeschke and Kokko, 2009; Del Giudice, 2014). Individuals who pursue a “fast” strategy tend to invest more in reproductive than in somatic effort, and more in mating than parenting effort, while individuals who pursue slow strategies tend to follow the reverse pattern (Figueredo et al., 2007). Fast strategies co-occur with behavioral traits such as “impulsivity, short-term thinking, promiscuity, low female parental investment, little or no male parental investment, little social support, disregard for social rules, and extensive risk-taking” (Figueredo et al., 2006). By contrast, slow strategies correlate with behavioral traits such as “long-term thinking, monogamy, extensive parental investment, substantial social support structures, adherence to social rules (e.g., cooperation and altruism), and careful consideration of risks” (Figueredo et al., 2006). Note that the term “strategy” is purely descriptive and does not imply conscious decision-making.

Seen this way, one may argue that within the spectrum of eating disorders individuals with BN are more likely to pursue fast life history strategies. This is supported by BN’s known association with heightened impulsivity, sensation seeking, novelty seeking and borderline personality disorder (Cassin and von Ranson, 2005), as well as with early menarche and early sexual experiences (Kaltiala-Heino et al., 2001). Conversely, individuals with AN may pursue slow life history strategies, compatible with their generally low impulsivity (at least in the restrictive subtype of AN; Waxman, 2009). Hence, the application of the concept of life history can help fine-tune the SCH where BN patients pursue a fast life history strategy and compete for short term mates through the display of immediate fecundity whereas AN patients pursue a slow life history strategy and compete for long-term mates through the display of thinness and hence reproductive potential (Del Giudice, 2014). It is, therefore, hypothesized that BN patients are engaged in future discounting and thus engaging in immediate reproductive effort whereas AN patients in their pursuit of a slow life history strategy are forgoing current reproduction in favor of doing so in the future (Del Giudice, 2018).

### Studies of Non-clinical Populations

Predictions from LHT and the SCH have been examined in a few non-clinical studies. Faer et al. (2005) were the first to explore whether and how intrasexual competition for mates, intrasexual competition for status and disordered eating behavior were connected to each other, predicting that intrasexual competition for mates would play a major role in BN, and intrasexual competition for status a major role in AN. In support of the SCH, intrasexual competition for mates appeared to be the determining factor for intrasexual competition for status, general competitiveness, perfectionism, drive for thinness, and body dissatisfaction. However, intrasexual competition for mates was also the determining factor for both anorexic and bulimic tendencies, whereas the effect of intrasexual competition for status on AN was only indirect through the influence of perfectionism.

Another study in female undergraduate students measured indicators of a “slow” life history strategy, disordered eating behavior, executive functions and female intrasexual competitiveness for mates and for status (Salmon et al., 2009). This work found an inhibitory effect of a “slow” life history strategy on disordered eating behavior as well as on female intrasexual competitiveness via executive functions as mediator, whereas no direct effect emerged of female intrasexual competition for mates and for status on disordered eating behavior. Instead, the model predicted that female intrasexual competitiveness and disordered eating behavior were spuriously correlated, with executive functions serving as the common causal influence on both. Similar results were found in a more recent study, demonstrating that a “slow” intrasexual competition strategy is negatively correlated with disordered eating behavior (Abed et al., 2012). Moreover, in line with Faer et al. (2005), this work reported that intrasexual competition is significantly related to disordered eating behavior (Abed et al., 2012). It is important to note that both of these studies relied on non-clinical college student samples, in which AN and BN could not be adequately distinguished. Therefore, the relative proportion of young women tending toward AN as opposed to BN in these sample is unknown, although one would consider it likely that low-level BN related behaviors would have a higher prevalence in these non-clinical populations.

In a further study, male and female participants were exposed to profiles of average attractive individuals with either high or low intrasexual competition cues. The high intrasexual competition cues led to more restrictive eating attitudes and body image concerns in heterosexual women and homosexual men, whereas homosexual women and heterosexual men showed no differences between the two conditions (Li et al., 2010). In addition, an investigation into the relationship between intrasexual competition for status and disordered eating behavior showed that exposure to thin, successful women resulted in greater body dissatisfaction in the participating women who scored high on status aspiration, than in the participating women with low scores on status aspiration (Smith et al., 2011). This suggests that intrasexual competition for status could play a role in the development of eating disorders independently of intrasexual competition for mates.

To the best of our knowledge no study has examined these hypotheses in clinical samples. Accordingly, we sought to examine predictions from SCH (including intrasexual competition for mates and status) and LHT in female patients with manifest AN and BN. Specifically we predicted that patients with AN and BN will score higher on measures for competition for mates and that patients with AN will score higher on measures for competition for status compared to controls. Also, we predicted that BN patients will show evidence of faster life history strategy on formal measurement compared to both AN and controls.

## Materials and Methods

### Participants

Sixty-nine women aged from 18 to 30 participated in this study of which 20 were diagnosed with AN and another 20 with BN according to DSM-IV criteria and a standardized diagnostic interview (see below). Twenty-nine female age-matched individuals formed a control-group. Individual body mass index (BMI) was calculated based on self-reported height and weight. The majority of patients were recruited from an in-patient unit and an outpatient setting of the Department of Psychosomatic Medicine, LWL University Hospital, Ruhr University Bochum. Controls were recruited from the local university. All participants received a small fee of 20 Euros for taking part in the study. The study was approved by the Ethics Committee of the Medical Faculty of the Ruhr-University Bochum, Germany (approval number 15-5298). The authors assert that all procedures contributing to this work comply with the ethical standards of the relevant national and institutional committees on human experimentation and with the Helsinki Declaration of 1975, as revised in 2008. All participants gave written informed consent. The demographic data of the three groups are summarized in Table 1.

**Table 1:** Demographic data.

	AN (*n* = 20)	BN (*n* = 20)	Control group (*n* = 29)	*p*-value
Age (years)	22.90 (3.78)	23.45 (2.72)	23.93 (2.34)	No significant differences
*Mean (SD)*	Range 18–30	Range 18–28	Range 20–29	
BMI (kg/m^2^)	15.27 (2.46)	22.43 (5.33)	22.17 (2.92)	AN significantly differs from BN and controls: *p* < 0.001
*Mean (SD)*				
Sexual orientation	90% heterosexual	75% heterosexual	93.10% heterosexual	
	5% bisexual	15% bisexual	3.45% homosexual	
	5% unspecified	5% homosexual		
		5% unspecified	3.45% unspecified	
Relationship status	75% single	75% single	68.97% in a relationship	
	20% in a relationship	20% in a relationship	20.96% single	
	5% married	5% other	6.90% married	
			3.45% other	


### Procedure

After giving informed consent, participants were screened for mental illnesses according to Mini-DIPS (Margraf, 1994) in order to gather information about psychiatric comorbid conditions in patients and to rule out psychiatric conditions in controls. Control cases indicating any past or present psychiatric disorders were excluded (Table 2).

**Table 2:** Screening for psychiatric comorbid conditions according to Mini-DIPS.

	AN (*n* = 20)	BN (*n* = 20)	Control group (*n* = 29)
Anxiety disorders	10	11	0
Obsessive-compulsive disorder	3	2	0
Affective disorders	9	13	0
Somatoform disorders	2	1	0
Eating disorders	20	20	0
Psychoactive substance use	1	1	0
Psychotic disorders	0	0	0


Subsequently, each participant received an individual anonymized participant code for the internet platform “eSurvey Creator”^1^, German version, and was asked to fill out several questionnaires including demographic data, the Eating Disorders Examination Questionnaire (EDEQ), the Arizona Life History Battery (ALHB), the Mate Value Inventory (MVI), the Female Intrasexual Competitiveness Scales for mates and status, respectively (ISCM and ISCS) and the Behavior Rating Inventory of Executive Function-Adult Version (BRIEF-A). All questionnaires were provided in German.

Participants were encouraged to fill out the questionnaires honestly and accurately. It was made clear that participating in this study would not influence further treatment of the patients.

### Measures

#### Mini-DIPS, Short Diagnostic Interview for Mental Disorders (Mini-DIPS, Diagnostisches Kurz-Interview bei Psychischen Störungen; Margraf, 1994)

The Mini-DIPS is a brief version of the German DIPS (Diagnostic interview for mental disorders) (Margraf et al., 1994). It is based on DSM-IV and ICD-10 criteria and facilitates a rapid assessment of the main psychiatric illnesses such as anxiety disorders and affective disorders. Eating disorders are included as well. As mentioned previously, the Mini-DIPS was used to confirm the respective diagnosis of eating disorders, to assess psychiatric comorbid conditions in patients and to rule out mental illnesses in controls.

#### Eating Disorders Examination Questionnaire (EDEQ; Fairburn and Beglin, 2008)

The EDEQ represents a self-report version of the Eating Disorders Examination interview (Cooper et al., 1989) and is utilized to record typical behavioral features and attitudes related to eating disorders. The questionnaire consists of 28 items revealing 4 subscales (Restraint, Shape Concern, Weight Concern, and Eating Concern) and a global score. High scores indicate higher degrees of disordered eating behavior. In the present paper, we used a German translation of the EDEQ constructed by Hilbert and Tuschen-Caffier (2006). It has been shown that the EDEQ has adequate internal consistency and test–retest reliability (Berg et al., 2012). Regarding validity, a recent study including a sample of 935 women with eating disorders found that the global score was quite efficient to discriminate between individuals with an eating disorder from those without an eating disorder (Aardoom et al., 2012), though the four subscales of the EDEQ were not supported by explorative factor analyses. As the mentioned study was the first to examine the validity of the EDEQ within such a large sample of individuals with eating disorders, we merely used the global score in our analysis. Higher scores indicate more disordered eating behavior.

#### Arizona Life History Battery (ALHB; Figueredo, 2007)

The ALHB is a battery composed of different original sources measuring cognitive and behavioral features that indicate an individual’s life history strategy. The component scales are “Mini-K Short Form,” “Insight, Planning, and Control,” “Mother/Father Relationship Quality,” “Family Social Contact and Support,” “Friends Social Contact and Support,” “Experiences in Close Relationships,” “General Altruism” and “Religiosity.” The subscale “Religiosity” was not included in the scoring of the ALHB due to differences in religiosity/secularism between Germany and the United States (Keller et al., 2013).

The majority of the component scales makes use of a 7-point Likert scale to specify how much the participants agree or disagree with each item. All items of the battery combined form the *K-Factor*, thus showing how much an individual corresponds to a slow (*K*-selected) LH strategy on the fast-slow (*r-K*) continuum. The internal consistency and measurement model structure of the ALHB have been reported to be adequate (Olderbak et al., 2014). Higher ALHB scores indicate a slower life history strategy.

#### Behavior Rating Inventory of Executive Function – Adult Version (BRIEF-A; Gioia et al., 2002)

As previous studies have demonstrated that the effect of life history strategies on psychosocial traits is at least partially mediated through executive functions (Figueredo et al., 2012; Wenner et al., 2013), we included the BRIEF-A self-report form to assess executive functioning of the participants. The BRIEF-A consists of 75 items that had to be scored in a range from 0 *(“never”)* to 6 *(“almost always”)* on a 7-point Likert scale. Two summary index scales [Behavioral Regulation Index (BRI) and Metacognition Index (MI)] and a global score (Global Executive Composite, GEC) were computed. The Behavioral Regulation Index (BRI) is a composition of 4 subscales (Inhibit, Shift, Emotional Control, and Self-Monitor), whereas the Metacognition Index (MI) is a composition of 5 subscales (Initiate, Working Memory, Plan/Organize, Task Monitor, and Organization of Materials). All subscales, the BRI, the MI and the global score were measured by building sum scores of the appropriate items with higher scores indicating lower executive functions. Ciszewski et al. (2014) found adequate reliability and validity of the BRIEF-A in a clinical sample with eating disorders (Ciszewski et al., 2014). Higher BRIEF-A scores suggest poorer executive functioning.

#### Female Intrasexual Competitiveness Scales (Faer et al., 2005)

The Intrasexual Competition for Mates Scale (ISCM) was used to measure intrasexual competition for mates and the Intrasexual Competition for Status Scale (ISCS) to measure intrasexual competition for status. Both are self-report questionnaires involving items that had to be rated according to the level of agreement in a range from 0 *(“strongly disagree”)* to 5 *(“strongly agree”)* and third-person vignettes about the fictional character Mary, whose behavior should be evaluated in a range from 0 *(“completely inappropriate”)* to 5 *(“completely appropriate”).* Items 3, 4, and 5 of the ISCS were scored inversely. High scores correspond to a high level of competitiveness in the measured domain. Internal consistency reliability was shown to be acceptable for both ISCM and ISCS (Faer et al., 2005). Higher values on the competition scales indicate greater competitiveness for mates or status.

#### Mate Value Inventory (MVI; Kirsner et al., 2003)

Participants completed the MVI-7 reporting self-perceived mate value. The form includes 17 items containing features which are desirable that had to be rated on a 7-point Likert scale from “*extremely low on this characteristic”* to “*extremely high on this characteristic*.”

The MVI has been shown to converge, among other factors, upon one common life history factor (Gladden et al., 2008; Abed et al., 2012), thus representing an indicator of life history strategy. High scores of the MVI indicate good confidence in oneself as a highly valuable potential mate.

The complete ALHB can be downloaded from Professor Figueredo’s homepage^2^. The other questionnaires can be obtained from the senior authors upon request by email.

### Statistical Analyses

Data analyses were performed using UniMult 2^3^. We used the conventional level of statistical significance by setting it at *p* < 0.05, but provided more exact probability values for any future meta-analytic purposes. Semipartial correlation coefficients are reported in the text, presenting both the magnitudes and direction of effects, with their statistical significance indicated by an asterisk after each corresponding parameter estimate; non-significant results are listed on the table but not reported in the main text as per APA guidelines.

Specifically, we applied a sequential canonical cascade model to these data based on previous work in similar domains (Gorsuch and Figueredo, 1991; Figueredo et al., 2016). The sequential canonical cascade model uses a pre-defined hierarchical organization of factors, whereby multiple dependent variables are fed into a sequential series of hierarchical regressions to predict the impact of each successive criterion variable, each of which taps into a specific aspect of life history relevant behaviors or attitudes, upon each of the subsequent ones. Accordingly, each prior criterion variable entered the equation as the first predictor for the next, such that each successive dependent variable is predicted from an initial set of pre-ordered predictor variables. This procedure allows to estimate the effect of each predictor (X) on each of the successive dependent variables (Y), while controlling for any indirect effects of the predictors through the prior dependent variables (for further details, see Figueredo et al., 2016). In summary, the sequential canonical cascade model is a statistical tool for identifying the magnitude of direct effects of independent variables on multiple correlated dependent variables. It is thus better suited for dimensional variables rather than categorical variables (such as diagnoses).

Following the implications of LHT on mating behavior, we hypothesized that a hierarchical order of factors made the most sense in the following sequence: (1) the overall score on the ALHB; (2) the global score of the executive functioning scale (BRIEF-A); (3) own mate self-reported value (MVI); (4) intrasexual competition for mates (ISCM); (5) intrasexual competition for status (ISCS); (6) disordered eating behavior (EDEQ). Specifically, the ranking regarding competition is based on one of the tenets of evolutionary theory, which gives primacy to the reproductive motivations over the social. Thus, intrasexual competition for mates is seen as more fundamental than intrasexual competition for status, and the latter is often seen as instrumental to obtaining the former, as higher status is believed to confer priority of access to a greater number and/or quality of sexual partners. This is often said of males but also probably applies to female dominance hierarchies.

## Results

### Group Comparisons

Data obtained in previous studies on non-clinical samples indicated that intrasexual competition, especially for mates, is associated with disordered eating behavior and that slow life history strategy may have a protective effect on disordered eating behavior. Our aim was to test how intrasexual competition and life history strategies bear upon clinically manifest eating disorders. Table 3 shows the global scores of the ALHB, BRIEF-A, MVI, ISCM, ISCS, and EDEQ as presented in our three different sample groups (AN, BN, and controls). Participants with AN and BN differed in several measures from the control group, partially corresponding to the findings of another recent study on larger sample sizes (Aardoom et al., 2012). Moreover, the mean BMI of the AN group was appreciably lower than the means of the BN group and the control group (see Table 1), suggesting that the clinical groups were fairly representative.

**Table 3:** Means and standard deviations (SD) for life history scores (ALHB), executive functioning (BRIEF-A), perceived own mate value (MVI), intrasexual competition for mates (ISCM) and status (ISCS) and disordered eating behavior (EDEQ).

Measure	AN (*n* = 20)	BN (*n* = 20)	Controls (*n* = 29)	*p*-values for planned comparisons
ALHB	1.25 (0.33)	0.88 (0.47)	1.44 (0.33)	AN > BN^∗^, C > BN^∗^
BRIEF-A	111.15 (58.12)	141.85 (62.24)	72.0 (34.23)	AN > C^∗^, BN > C^∗^
MVI	0.94 (0.57)	0.52 (0.82)	1.11 (0.81)	AN < C^∗^, BN < C^∗^
ISCM	1.32 (0.79)	1.55 (0.68)	0.93 (0.62)	BN > C^∗^
ISCS	1.34 (0.59)	1.53 (0.66)	1.47 (0.52)	n.s.
EDEQ	3.50 (1.36)	3.97 (1.15)	0.73 (0.62)	AN > C^∗^; BN > C^∗^


*Higher ALHB scores indicating slower life history; higher BRIEF-A values indicating poorer executive functioning; Higher MVI values indicating greater perceived own mate value; larger figures for intrasexual competition and status indicating greater competitiveness; higher values for eating behavior representing more disordered eating. ^∗^p < 0.05.*

### Correlation Analysis

Table 4 shows correlation coefficients for those variables that were chosen for the subsequent sequential canonical analysis. As expected, AN and BN correlated with BMI and scores of the EDEQ. Additional unique correlations emerged for BN with life history measures, executive functioning and mate value scores. The ALHB score also correlated with executive functioning (BRIEF), mate value, intrasexual competition for mates, and eating disorder. Together, these findings suggest that people with BN displayed signs of a fast life history strategy, whereas individuals with AN did not differ from controls on most life history scores, except in executive functioning and perceived mate value, as discussed below.

**Table 4:** Correlation **c**oefficients.

Variables	Age	BMI	AN	BN	ALHB	BRIEF-A	MVI	ISCM	ISCS
Age									
BMI	0.16								
AN	–0.13	–0.66*							
BN	–0.01	0.29*	–0.41*						
ALHB	–0.10	–0.16	0.04	–0.49*					
BRIEF-A	0.18	0.03	0.08	0.42*	–0.68*				
MVI	–0.01	0.02	–0.13	–0.47*	0.69*	–0.61*			
ISCM	–0.11	–0.02	0.09	0.29*	–0.49*	0.27*	–0.23		
ISCS	0.01	0.13	–0.12	0.09	–0.22	0.21	–0.11	0.55*	
EDEQ	–0.09	–0.16	0.36*	0.53*	–0.55*	0.63*	–0.65*	0.36*	0.05


*^∗^p < 0.05.*

### Sequential Canonical Analysis

The multivariate test for the entire sequential canonical analysis model was statistically significant (Pillai-Bartlett *V* = 0.686, η = 0.37, *F* = 27.58, *p* < 0.0001), indicating an omnibus protective test of overall statistical significance. Table 5 displays the results of this analysis.

**Table 5:** Multivariate hierarchical (sequential) regression analysis for the criterion variables.

Criterion variables	Prior criterion variables	Effect size (CI)	*F*-ratio	*df1/df2*	*p*
BRIEF-A					
	ALHB	*r* = –0.68 (–0.79,–0.52)	57.19	1/67	<0.0001
MVIself					
	BRIEF	*r* = –0.61 (–0.74,–0.43)	49.10	1/66	<0.0001
	ALHB	*r* = 0.35 (0.12,0.55)	16.29	1/66	<0.0001
ISCM					
	MVIself	*r* = –0.23 (–0.44,0.02)	3.98	1/65	0.05
	BRIEF	*r* = 0.16 (–0.08,0.39)	2.10	1/65	0.15
	ALHB	*r* = –0.29 (–0.50,–0.05)	6.59	1/65	0.01
ISCS					
	ISCM	*r* = 0.55 (0.35,0.70)	27.90	1/64	<0.0001
	MVIself	*r* = 0.01 (–0.23,0.25)	0.01	1/64	0.91
	BRIEF	*r* = 0.09 (–0.15,0.33)	0.79	1/64	0.38
	ALHB	*r* = 0.08 (–0.16,0.32)	0.61	1/64	0.44
EDEQ					
	ISCS	*r* = 0.05 (–0.20,0.29)	0.35	1/63	0.38
	ISCM	*r* = 0.40 (0.18,0.59)	25.27	1/63	<0.0001
	MVIself	*r* = –0.58 (–0.72,–0.40)	53.01	1/63	<0.0001
	BRIEF	*r* = 0.28 (0.04,0.48)	11.72	1/63	0.001
	ALHB	*r* = 0.10 (–0.14,0.34)	1.63	1/63	0.21


Poor executive functioning (BRIEF-A, which is reverse-scored to indicate difficulties) was significantly predicted to decrease by the life history (ALHB) factor, suggesting that a slower life history was associated with better executive functioning (-0.68^∗^).

As regards own mate value (MVI), superior executive functioning predicted an increased self-perceived mate value (-0.61^∗^). Moreover, slower life history also directly and significantly predicted an increase in self-rated mate value (0.35^∗^).

Intrasexual competition for mates (ISCM) was predicted to decrease by own mate value ratings (-0.23^∗^), that is, higher perception of one’s own mate value predicted a decreased intrasexual competition for mates. Intrasexual competition for mates was also directly and significantly predicted to decrease by a slower life history (-0.29^∗^), but was not directly and significantly predicted by poor executive functioning. Intrasexual competition for status (ISCS), in contrast, was only directly and significantly predicted by an increase by intrasexual competition for mates (0.55^∗^) and showed no other significant direct effects upon that construct.

Finally, disordered eating behavior (EDEQ) was directly and significantly predicted to increase by intrasexual competition for mates (0.40^∗^), but not for status, and it was also directly and significantly predicted to increase by poor executive functioning (0.28^∗^), but was not further directly and significantly predicted by slower life history scores. Disordered eating behavior was directly and significantly predicted to decrease by higher self-perceived mate value (-0.58^∗^).

Reported confidence intervals (CIs) were the lower and upper bounds for an approximate 90% level of confidence, uncorrected for the number of other variables included in the model. Criterion variables were cascaded, with the prior criterion variables partialled out in reverse order for the path analytic interpretation of direct and indirect effects. All regression residuals were evaluated for normality of distribution, and both the skewness and kurtosis parameters were found to be within conventional limits (-1.96,+1.96) for each criterion variable tested.

## Discussion

This study set out to test predictions of the evolutionarily-based SCH that states that eating disorders arise from abnormally intense female intra-sexual competition as a result of mismatch with the conditions of the modern western and westernized urban environment. We also aimed to test the predictions of a further evolutionary idea namely that life history strategies shape the clinical features and manifestations of AN and BN, with AN tending to be on the slow side and BN on the fast side of the life history continuum.

Our predictions were partially confirmed. For example, as outlined above and at the end of the introduction section, we found that BN was associated with several measures typical for a fast life history strategy, whereas AN did not significantly differ from controls in this regard. Implicitly, this suggests that individuals with AN lie on the slow end of the life history continuum, based on the assumption that the control sample consisting mainly of college students pursued a slow life history strategy. However, there was no direct causal relationship between disordered eating behavior and life history strategies as measured using the EDEQ and the ALHB. These findings are in partial contrast to previous studies examining life history strategies in connection with disordered eating behavior, which reported an inhibitory effect of a slow life history strategy on disordered eating behavior, meaning that eating disorders would be rather associated with a fast life history strategy (Salmon et al., 2009; Abed et al., 2012). A plausible explanation for this result could be that both previous studies did not clearly differentiate between AN and BN, due to the lack of formal clinical diagnoses in these non-clinical populations. Furthermore, Salmon et al. (2009), who used the Eating Disorders Inventory (EDI-2) to assess disordered eating behavior, reported that their sample scored higher on the bulimia subscale, as compared to previous data from a clinical anorexic restricting sample and a college non-anorexic sample. The non-clinical sample of Abed et al. (2012), on the other hand, engaged in more objective binge eating episodes and excessive exercise compared to data from two other non-clinical samples. In addition, we know that the prevalence of BN is at least three times greater than AN in community surveys (Hoek and van Hoeken, 2003). Hence the much higher levels of bulimia scores and binge eating behaviors in previous non-clinical studies together with the higher prevalence of BN in community samples would help explain the previous findings of eating disorders being associated with a fast life history strategy and the suggested protective role for slow life history strategy. In addition, while we used the composite score of the EDEQ for statistical analyses, we cannot rule out that the inclusion of the “restraint” subscale biased our findings in some way, because “restraint” is more typical for AN, rather than BN.

In any event, the present study is the first to test concepts of LHT within a clinical sample of AN and BN patients, thus being clearly grounded in an evolutionary framework. Our findings show that not only a fast, but also a slow life history strategy can be associated with disordered eating behavior although in different ways.

In addition, we discovered a correlation between slower life history strategies and higher self-perceived mate value when pooling the data of all three groups, which lends further support to the assumption that high mate value serves as an indicator of a slow life history strategy (Abed et al., 2012). Accordingly, as the AN group emerged to pursue a slow life history strategy, we expected individuals with AN to show high levels of self-perceived mate value. In fact, slightly higher self-perceived mate value than average has been already found in young college women with tendencies toward anorexic behaviors related to the drive for thinness (Faer et al., 2005). However, our data showed lower self-perceived mate value for both AN and BN compared to controls. This lower scoring in the AN and the BN group could be attributed to low self-esteem, which has been found to be associated with clinically manifest eating disorders in general (Gual et al., 2002).

We corroborated previous work suggesting that preserved executive functioning correlated with a slow life history strategy (Salmon et al., 2009; Figueredo et al., 2012), as slower life history scores significantly predicted higher executive functioning. Moreover, better executive functioning also predicted less disordered eating behavior.

Interestingly, in our study both patients with AN and BN performed more poorly in executive functions than controls, which is in agreement with prior work (Salmon et al., 2009; Tchanturia et al., 2012), but inconsistent with predictions from LHT. According to LHT, a slow life history strategy in AN should be associated with preserved executive functions, whereas a fast life history strategy in BN should go along with poorer executive functions. The fact that we found poor executive functioning in patients with AN could suggest that women with manifest AN display poor executive functions secondary to somatic problems that arise from dietary restriction. Several studies have revealed that AN is associated with brain volume reductions, depending on the duration and severity of the condition, whereas these abnormalities do not occur to the same extent in patients with BN (Brooks et al., 2011; Jáuregui-Lobera, 2011; Seitz et al., 2016; Martin Monzon et al., 2017). Notably, a recent study reported that adolescent AN patients have decreased gray matter volume in the inferior frontal gyrus, which may lead to deficits in executive functioning (Fujisawa et al., 2015).

We found that intrasexual competition for mates, but not status, was significantly predicted by one’s own perceived mate value and by other signs of a fast life history strategy. That is, participants who perceived their own mate value as low engaged in greater competition for mates. Consistent with this finding, low scores on the ALHB indicating a faster life history strategy significantly predicted more intense intrasexual competition for mates. Group comparisons revealed that this strategy was apparently more relevant for individuals with BN, but not AN, which is, at least in part compatible with findings in non-clinical samples (Faer et al., 2005; Li et al., 2010; Abed et al., 2012).

Together, our study provides first insights from LHT by examining how intrasexual competition and other markers of slow and fast life history strategies are linked to clinically manifest eating disorders. In particular, our results revealed that individuals with a relatively faster life history strategy have poorer executive functioning, lower own mate values, and more intense intrasexual competition for mates, but not status. Overall, in our sample these findings are more convincing for people with BN whereas those with AN seem to be more similar to controls in terms of life history strategy, except executive functioning, as discussed above.

## Limitations and Future Research

The present study has several limitations. First, the relatively small sample size was at best suitable for an explorative analysis of the relationship between life history strategies, intrasexual competition and clinically manifest eating disorders, but needs to be replicated in larger samples. Second, for the same reason we could not take into account statistically differences in patterns of comorbid psychiatric conditions such as anxiety disorders and affective disorders. Third, the control group consisted mainly of college students, which might have caused a bias in the analysis toward slower life history strategies in this group. Another limitation is that that our questionnaires, as well as information regarding height and weight, were based on self-assessment and therefore susceptible to response biases. Furthermore, it remains unclear, if the association of life history strategy with eating disorders is based on trait-related issues or state-related issues (e.g., altered BMI). That is, an individual’s life history strategy may change due to weight gain or loss and with changes in physical health status.

As we wanted to include primarily women in early stages of the disease and at the peak of their reproductive potential, the age range was restricted to 18 to 30 years. Consequently, we did not capture younger patients and therefore especially missed patients with AN, which is known to often have an age of onset around puberty or early adolescence (Volpe et al., 2016). Likewise, we did not capture male patients considering that mainly women are affected by eating disorders. Given that Li et al. (2010) found cues of high intrasexual competition leading to greater body image dissatisfaction and eating restriction not only in heterosexual women, but also in homosexual men, further investigations may also include male homosexual patients with eating disorders.

Besides, it would be interesting to assess patients before and after treatment to account for trends concerning intrasexual competition, to determine whether intrasexual competition changes in response to therapy.

While there are a number of rival evolutionary theories for AN (other than SCH) there are very few aimed specifically at explaining BN and none that are currently supported by empirical evidence. Hence this study is the first to find evidence for increased intrasexual competition for mates in patients with a clinical diagnosis of BN. Moreover, the whole sample of eating disorder patients (AN and BN combined) showed significantly increased intrasexual competition for mates with higher EDEQ scores, thus lending further support to the SCH in a clinical population with eating disorders.

Hence, in addition to the small sample size already referred to, our failure to detect higher intrasexual competition for mates in AN may have other explanations. These include: (1) contrary to SCH, AN, and BN may have distinct evolutionary roots; or (2) the current classifications of eating disorders may not reflect the deep structure of these disorders (Del Giudice, 2018).

Finally, our findings may be utilized in psychotherapy insofar, as it may be warranted to reflect with patients upon the roots, origins and content of their unconscious decision-making, self-evaluation and mating behavior, to reduce the potentially harmful effects that may arise from engaging in intense and harmful competition and/or living a life “fast and furious” (Brüne, 2016).

## Author Contributions

JN contributed to acquisition, analysis, and interpretation of the data, drafted the article, and approved the final version to be submitted. GG interpreted the data, revised the manuscript for intellectual content, and approved the final version to be submitted. SH designed the study, interpreted the data, revised the manuscript for important intellectual content, and approved the final version to be submitted. RA designed the study, interpreted the data, drafted the article, revised the manuscript for important intellectual content, and approved the final version to be submitted. AF designed the study, contributed to analysis and interpretation of the data, revised the manuscript for important intellectual content, and approved the final version to be submitted. MB design the study, contributed to analysis and interpretation of the data, drafted the article, revised the manuscript for important intellectual content, and approved the final version to be submitted. All authors have approved the final article.

## Conflict of Interest Statement

The authors declare that the research was conducted in the absence of any commercial or financial relationships that could be construed as a potential conflict of interest.

## Footnotes

1 https://www.esurveycreator.com

2 https://sites.google.com/email.arizona.edu/ajf

3 http://www.unimult.com/
